# The Gene Sculpt Suite: a set of tools for genome editing

**DOI:** 10.1093/nar/gkz405

**Published:** 2019-05-25

**Authors:** Carla M Mann, Gabriel Martínez-Gálvez, Jordan M Welker, Wesley A Wierson, Hirotaka Ata, Maira P Almeida, Karl J Clark, Jeffrey J Essner, Maura McGrail, Stephen C Ekker, Drena Dobbs

**Affiliations:** 1 Bioinformatics and Computational Biology Program, Iowa State University, Ames, IA 50011, USA; 2 Genetics, Development and Cell Biology Department, Iowa State University, Ames, IA 50011, USA; 3 Department of Physiology and Biomedical Engineering, The Mayo Clinic, Rochester, MN 55905, USA; 4 Department of Biochemistry and Molecular Biology, The Mayo Clinic, Rochester, MN 55905, USA

## Abstract

The discovery and development of DNA-editing nucleases (Zinc Finger Nucleases, TALENs, CRISPR/Cas systems) has given scientists the ability to precisely engineer or edit genomes as never before. Several different platforms, protocols and vectors for precision genome editing are now available, leading to the development of supporting web-based software. Here we present the *Gene Sculpt Suite* (GSS), which comprises three tools: (i) GTagHD, which automatically designs and generates oligonucleotides for use with the GeneWeld knock-in protocol; (ii) MEDJED, a machine learning method, which predicts the extent to which a double-stranded DNA break site will utilize the microhomology-mediated repair pathway; and (iii) MENTHU, a tool for identifying genomic locations likely to give rise to a single predominant microhomology-mediated end joining allele (PreMA) repair outcome. All tools in the GSS are freely available for download under the GPL v3.0 license and can be run locally on Windows, Mac and Linux systems capable of running R and/or Docker. The GSS is also freely available online at www.genesculpt.org.

## INTRODUCTION

**Figure 1.**
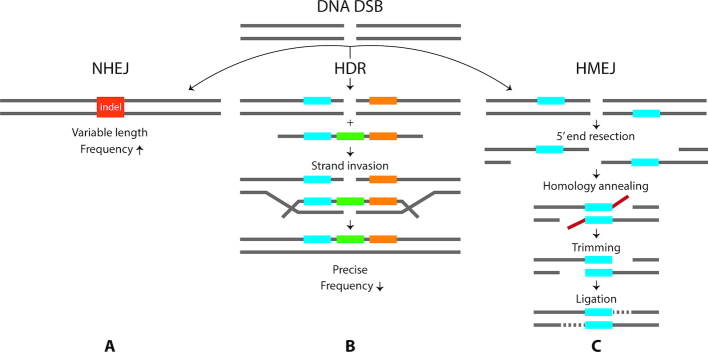
DSB repair mechanisms. (**A**) NHEJ. The DNA DSB ends are bound by the Ku70–Ku80 heterodimer and undergo limited end-resection before DNA polymerases and ligases repair the break. This process may perfectly repair the DSB break, but more frequently introduces short indels (red). (**B**) HDR. When a DSB is detected, homologous sequences (blue and orange segments), frequently provided by a sister chromatid are used as a template to repair the break (green). The resulting repair is usually precise. (**C**) HMEJ. HMEJ is a catch-all term for repair that utilizes short regions of homology, including MMEJ and SSA. In both MMEJ and SSA, 5′-3′ end-resection exposes single-stranded DNA regions, where homologous sections (blue) anneal with one another for repair. The overhanging DNA strands (red) are then clipped, resulting in a short deletion. MMEJ and SSA are mechanistically similar but distinct pathways, utilizing different protein machinery. MMEJ also utilizes shorter regions of microhomology (∼2–25 bp) than SSA (>25 bp). SSA end-resection can be extensive, so the pathway operates over larger nucleotide distances.

Recent additions to the gene editing toolbox include methods for identification of off-target sites (1,2), strategies for improving nuclease specificity (3) and the expansion of nuclease targeting capabilities (4–7). Other approaches have focused on DNA double-strand break (DSB) repair by increasing the efficiency of homology-directed repair (HDR)/homologous recombination (HR) or enhancing the precision of the non-homologous end joining (NHEJ) DNA repair pathway (8) (see Figure 1A and B). However, relatively little work has been done to leverage homology-mediated end joining (HMEJ) pathways (Figure 1C), including microhomology-mediated end joining (MMEJ) and single-strand annealing (SSA), and their potential to enhance the efficiency, precision and reproducibility of gene-editing experiments.

Gene knock-in research has focused on increasing the frequency of HDR/HR-based DSB repair to precisely integrate DNA cargo into a genomic locus, e.g. by modifying the Cas9 protein (9) or inhibiting NHEJ (10). However, these methods can be difficult to implement and can be highly inefficient, with only a few successful knock-ins per hundreds of attempts. In addition, HR is almost completely inhibited during the G1 phase of the cell cycle (11), which inhibits targeted integration in post-mitotic cells and decreases gene-editing knock-in efficiencies in embryos. Much of the recent research on enhancing gene knockouts has focused on NHEJ. This pathway has been thought to repair DNA DSBs in an apparently random and inherently error-prone fashion through the introduction of short indels. Recent work has demonstrated that these errors are not necessarily random and are frequently reproducible (12–14). Although there are now methods for predicting repair profiles (12,13), DSB sites that rely heavily on NHEJ—as opposed to MMEJ—often lead to highly mosaic DSB repair profiles, i.e., they do not display a single predominant repair outcome (12).

In contrast, the Gene Sculpt Suite (GSS) tools (GTagHD (15), MEDJED, and MENTHU (16)) leverage HMEJ, a catch-all term for repair methods such as MMEJ and SSA, which utilize short regions of sequence homology to repair DSBs. GTagHD aids researchers in implementing the GeneWeld protocol, which leverages HMEJ repair to introduce targeted knock-ins with efficiencies much higher than previously reported (15). MMEJ repairs frequently have highly predictable outcomes based on the ‘strength’ of the microhomology regions present (17). The relative strengths of these homologies can be used to identify predominant MMEJ allele (PreMA) reagents, i.e., nucleases that target sites likely to result in a single MMEJ-based deletion composing >50% of all repair outcomes (16). MENTHU and MEDJED are GSS tools designed to assist researchers in identifying PreMA reagents (16) and assessing the MMEJ potential of potential target sites, respectively.

## RESULTS

### Availability and implementation

**Figure 2.**
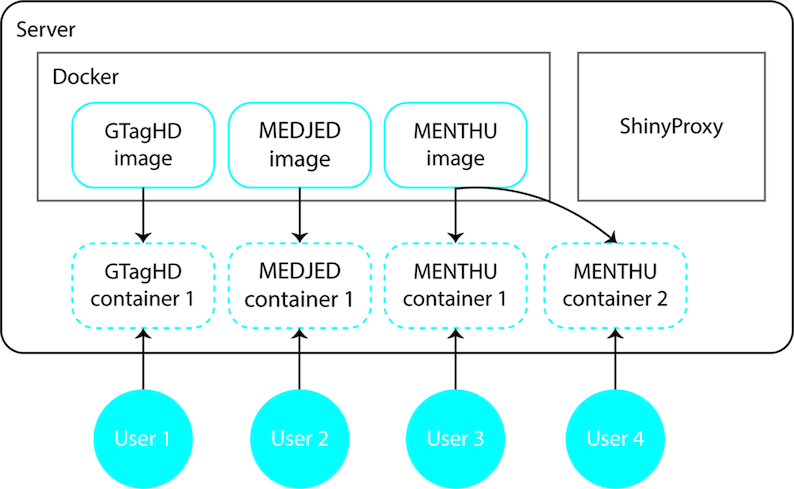
GSS Architecture. The GSS server uses ShinyProxy (https://www.shinyproxy.io/) to administer the Docker images (solid blue line) for each GSS tool. When a user (blue circle) visits a GSS tool URL, ShinyProxy creates a Docker container (dashed blue line), which essentially is a temporary copy of the Docker image and allows a user to securely interact within their own container. These containers are temporary, and deleted once a user leaves their URL. A new container is spun up for each unique user.

The GSS server is hosted on an Amazon Web Services Elastic Compute Cloud Ubuntu 16.04 LTS instance. Each tool was built in R (https://www.r-project.org/) using RStudio (https://www.rstudio.com/) and is an RShiny (https://shiny.rstudio.com/) application contained in a Docker (https://www.docker.com) image using the Open Analytics r-base image (https://hub.docker.com/r/openanalytics/r-base). When a user visits a GSS tool URL, ShinyProxy (https://www.shinyproxy.io) spins up a new container from that tool's Docker image; the user can then securely interact within the confines of their container until they close their browser page (Figure 2). ShinyProxy releases and deletes the container one minute after the browser connection has closed. This allows users to securely interact with the server in their own virtual environments.

Each tool in the Suite is also available for download via GitHub (https://github.com/Dobbs-Lab) and as a Docker image through Docker Hub (https://hub.docker.com/u/cmmann). These tools can be run locally on Windows, Linux and Mac operating systems capable of running R v3.5.2 or later and/or Docker v18.06.1-ce or later. All tools are available at www.genesculpt.org, which also includes links to the GitHub and Docker Hub repositories.

### GTagHD

**Figure 3.**
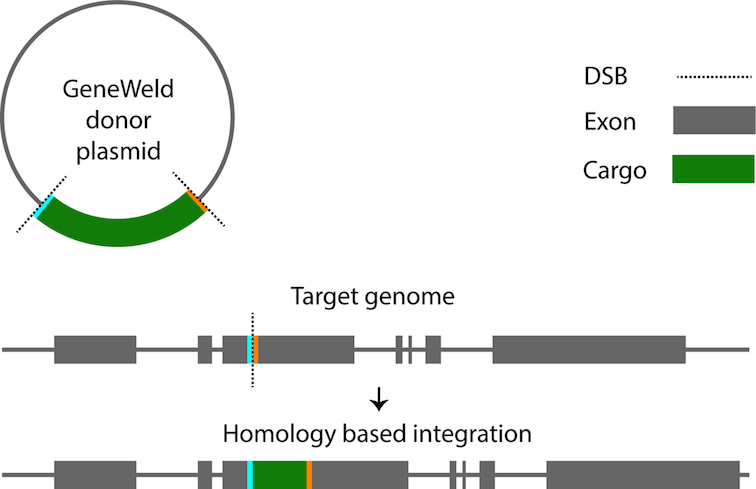
GeneWeld integration scheme (15). Short homologous sequences from the integration site in the target genome (in blue and orange) are cloned into the flanking regions of the donor plasmid cargo (green). When the cargo is freed from the plasmid, the homologous regions promote the efficient and precise integration of the cargo into the genomic locus using HMEJ. The plasmid and genomic DNA DSBs are generated by two separate gRNAs.

GTagHD (p**GT**ag **H**omology **D**esigner) designs oligonucleotides for use with the GeneWeld protocol ((15); see Figure 3). GeneWeld uses short sections of sequence homology between a plasmid donor and a genomic locus to efficiently and precisely integrate the plasmid cargo into the specified locus, with minimal disruption to surrounding DNA. For additional details regarding the GeneWeld technology and its advantages over previous integration methods see Wierson et al. (15).

#### Input

GTagHD takes the genomic integration site with surrounding DNA sequence and a user-specified length of sequence homology between the plasmid donor and integration site as input. Users input the genomic locus as a pasted DNA sequence or GenBank, RefSeq or Ensembl ID. The gRNA sequence used to target the integration site is input as the 20-nt guide (with no Protospacer Adjacent Motif (PAM) sequence). GTagHD assumes a Cas9-like DSB will be generated 3 bp upstream of the PAM sequence, allowing flexibility in the choice of CRISPR nuclease in targeting the genomic locus. We have developed two plasmid series for use with the GeneWeld protocol, and although we strongly recommend using these plasmids with GTagHD, the tool also supports custom plasmids and cargos, which require the gRNA sequence for freeing the cargo from the custom plasmid as the only additional input.

#### Processing

GTagHD identifies the integration site using the provided genomic gRNA sequence. GTagHD checks to ensure that this gRNA appears exactly once within the provided genomic DNA, but does not check for off-target sites within the rest of the genome; several tools (including CRISPRscan (18)) are available for this purpose. GTagHD extracts the user-specified length of homologous sequence surrounding the integration site, automatically adds additional nucleotides to repair frameshifts caused by the DSB, adds restriction enzyme sites for cloning into the plasmid, accounts for custom plasmid gRNAs (if provided) and performs additional plasmid-series dependent processing.

#### Output

GTagHD outputs four oligonucleotide sequences: 5′ ‘forward’, 5′ ‘reverse’, 3′ ‘forward’ and 3′ ‘reverse’. The oligonucleotides sequences can be downloaded as a text file and are ready-to-order. The synthetic oligonucleotides can be easily cloned into a plasmid vector. If a user chooses to use a plasmid from the GeneWeld series, they can also download automatically-generated plasmid maps containing their incorporated oligonucleotides in A Plasmid Editor (ApE) format, which is compatible with the GenBank format (gb).

#### Comparison to other methods

The *GeneWeld* protocol was inspired by the PITCh protocol (19–20), which is also available for designing knock-in construct guides (http://www.mls.sci.hiroshima-u.ac.jp/smg/PITChdesigner/index.html). However, GTagHD has a few features that may make it more convenient for users than the PITCh designer 2.0 webtool (21).

First, users can submit GenBank, RefSeq and Ensembl IDs to specify their genomic locus, instead of copying and pasting whole sequences as in PITCh 2.0. When using an ID, GTagHD can automatically identify and repair frameshifts created by the DSB site to maintain the correct codon and keep the original sequence in frame and intact. PITCh 2.0 requires users to manually specify the reading frame and corrects frameshifts by inserting ‘Cs’ or by deleting a codon entirely, thus altering the original genomic sequence.

Second, GTagHD identifies the DSB integration site in the genomic sequence from user-provided gRNA, and does not require users to manually scroll through the sequence to identify the location, as in PITCh 2.0.

Finally, GTagHD does not require any information about the plasmid vector beyond (possibly) the gRNA sequence used to free the cargo, whereas PITCh 2.0 requires sequence context from the insert.

### MEDJED

**Figure 4.**
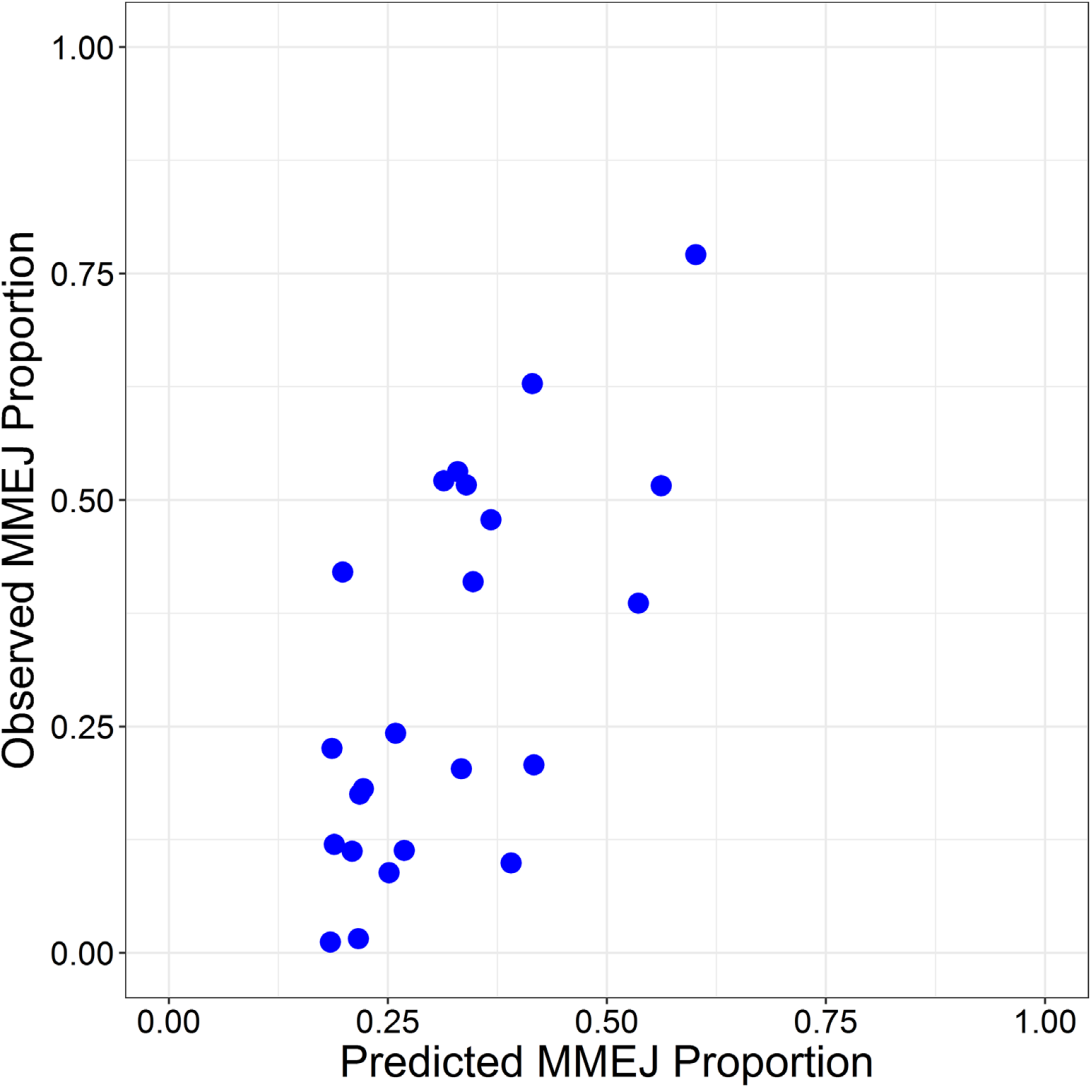
MEDJED performance. On a test set of 23 HeLa cell targets from (17), MEDJED achieves a Pearson Correlation Coefficient (PCC) of 85.2%, MAE of 10.3% and Root Mean Square Error (RMSE) of 12.0%. The MEDJED-predicted MMEJ repair proportion (*x*-axis) is graphed against the observed MMEJ repair proportion (*y*-axis).

MEDJED (**M**icrohomology **E**voked **D**eletion **J**udication **E**luci**D**ation) is a random forest machine learning-based method for predicting the extent to which a DSB site will undergo MMEJ repair. MEDJED was trained on 66 and tested on 23 CRISPR Cas9 sites in HeLa cells acquired from Bae *et al.* (17). As shown in Figure 4, when comparing the predicted proportion of MMEJ-based deletions against the observed proportion of MMEJ-based deletions on an independent test set, MEDJED achieved a correlation coefficient of 85.2%, mean absolute error (MAE) of 10.3%, and root mean square error (RMSE) of 12.0%.

#### Input

MEDJED takes a pasted DNA sequence between 20 and 200 nt in length as input and assumes the DSB occurs in the exact middle of the sequence.

#### Processing

MEDJED assesses the strengths of all microhomologies present, utilizing features including the minimum deleted sequence length, the maximum, mean and standard deviation of the microhomology arm lengths, and the maximum and standard deviation of the Microhomology–Predictor pattern score (17). These features are input into the MEDJED regression model.

#### Output

MEDJED returns a prediction of the proportion of deletion repair outcomes at the provided site expected to result from MMEJ-based repair. It also outputs the values of the six features used in predicting the MMEJ-based repair proportion, as well as a table of all the MMEJ-based deletion outcomes for the targeted site. These outputs can be downloaded individually or collectively as a zip file.

#### Comparison to other methods

The Microhomology–Predictor (http://www.rgenome.net/mich-calculator/, (17)), on which MEDJED is partially based, calculates an ‘out-of-frame’ score for choosing DSB sites likely to generate out-of-frame deletions; if the score is above 66, the site is recommended for generating gene knockouts. Microhomology–Predictor does not, however, predict the extent of MMEJ at a particular site, and while the out-of-frame score tends to correlate closely with the observed proportion of out-of-frame repairs, it is not a probability of such events occurring.

inDelphi (https://indelphi.giffordlab.mit.edu/, (13)) and FORECasT (Favoured Outcomes of Repair Events at Cas9 Targets, https://partslab.sanger.ac.uk/FORECasT, (12)) both predict expected ‘repair profiles’ at a DSB site—that is, they enumerate all possible repair outcomes for a particular site (within a limited sequence window), and compute the probability of each outcome. inDelphi is notably feature-rich and offers the option to predict probabilities in different cell types; however, determining the probability of MMEJ-based repair for a particular site requires additional calculations on the part of the user. FORECasT, while simple to use, does not output an intuitive human-readable result, requiring users to perform remapping of each outcome to calculate the predicted proportion of MMEJ repair.

### MENTHU

MENTHU (**M**icrohomology-mediated **E**nd joining k**N**ockout **T**arget **H**euristic **U**tility) identifies sites likely to have a predominant microhomology-mediated end joining allele (PreMA) repair outcome (16). MENTHU expands on the Microhomology–Predictor tool algorithm (17), which produces a ‘pattern score’ for each possible MMEJ-based deletion within a sequence. This score is based on the length, GC content and deleted sequence length expected to be produced by the microhomology, with a higher score corresponding to a ‘stronger’ microhomology. MENTHU evaluates the ratio between the two highest scoring deletions as a surrogate for relative competitiveness between microhomology sites in recruiting the MMEJ machinery, in order to identify ‘low competition’ sites where a single microhomology pairing is likely to be predominant. For additional details, see Ata et al. (16).

#### Input

MENTHU takes a user-specified CRISPR or TALEN nuclease and a target DNA region as input. Users can choose from a list of CRISPR nucleases or can specify custom nucleases by providing a PAM sequence, distance between DSB site and PAM, and length of 5′ overhangs (for nucleases producing sticky-end DSBs, like Cas12a). The genomic DNA target can be specified by pasting a DNA sequence or a GenBank, RefSeq or Ensembl ID. MENTHU also allows users to specify exons to increase search speed and biological relevance of the results.

#### Processing

MENTHU scans the input DNA for selected nuclease target sites. For each matching site, MENTHU identifies all microhomology pairings within an 80 bp window centered at the DSB site and then scores them according to the algorithm employed by Microhomology–Predictor (17). MENTHU then identifies sites in which the highest scoring predicted deletion has ≤5 intervening nucleotides between the microhomology arms in the wild type sequence and calculates the quotient between its pattern score and the next highest scoring microhomology. This ratio is the MENTHU score.

#### Output

**Figure 5.**
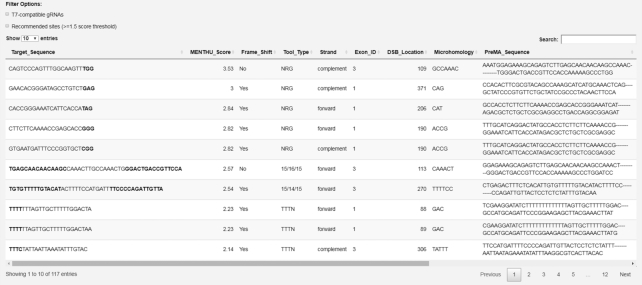
Example MENTHU output table. Each row corresponds to a single DSB event. The ‘Target_Sequence’ column contains the gRNA or TALEN sequence required to generate the DSB. The ‘MENTHU_Score’ column gives the ratio between the Microhomology–Predictor pattern scores of the top two scoring microhomologies at the site; a DSB site is likely to produce a PreMA if the MENTHU Score is ≥1.5 (16). ‘The Frame_Shift’ column indicates whether the most frequent expected deletion pattern induces a frameshift. The ‘Tool_Type’ gives the PAM sequence for CRISPR nucleases, and the left arm length/spacer/right arm length combination for TALENs. The ‘Strand’ column indicates whether the ‘Target_Sequence’ occurs on the forward or complement strand. The ‘Exon_ID’ provides the number of the exon in which the DSB site occurs; if no exon information is available, this value is 1. The ‘DSB_Location’ provides the index of the nucleotide to the left of the DSB site within the entire nucleotide sequence. The ‘Microhomology’ column contains the sequence of the microhomology arms used to generate the deletion. The ‘PreMA_Sequence’ gives the sequence of the predicted predominant repair outcome. The ‘Context’ column (not shown) gives the sequence window used for MENTHU score calculations.

MENTHU outputs a table of likely PreMA reagents in descending order of MENTHU score (Figure 5). The table consists of ten columns. The ‘Target_Sequence’ provides the gRNA or TALEN sequence needed to induce a DSB at a particular site. The ‘MENTHU_Score’ column contains the computed MENTHU score. The ‘Frame_Shift’ column indicates whether the PreMA deletion generates a frameshift. The ‘Tool_Type’ provides the PAM sequence, in the case of CRISPR nucleases, and the length of the arms and spacer in the case of TALEN inputs. The ‘Strand’ column indicates whether the Target_Sequence matches the forward or complement strand. The ‘Exon_ID’ gives the exon in which the Target_Sequence site occurs, while the ‘DSB_Location’ gives the position of the nucleotide directly to the left of the DSB site. The ‘Microhomology’ column gives the sequence of the microhomology producing the deletion. The ‘PreMA_Sequence’ column shows the top predicted MMEJ deletion sequence (PreMA) for the site. The ‘Context’ column (not shown) gives the ‘wildtype’ sequence corresponding to the PreMA region. The table is searchable, sortable, and can be downloaded in CSV format. Targets can be filtered to show only recommended sites (with MENTHU score > 1.5). By default, all sites for which the top MMEJ deletion has ≤5 bp between microhomology arms in wild type sequence are shown, although the results can be filtered to show only recommended sites (MENTHU score ≥ 1.5). Targets can also be filtered to display only T7-compatible gRNAs.

#### Comparison to other methods

The Microhomology–Predictor tool (17), FORECasT (12) and inDelphi (13) all assist users in choosing sites for gene knockout. However, MENTHU has several key features that may make it more convenient for some users. MENTHU utilizes the Pattern Score devised by Bae *et al.* and used in the Microhomology–Predictor tool (17). As previously described, the Microhomology–Predictor uses the Pattern Score to identify sites likely to produce a frameshift (and by extension, gene knockout). In contrast, MENTHU uses the ratio between Pattern Scores for various MMEJ-based deletion patterns to approximate ‘competition’ between available microhomologies for use by the MMEJ repair machinery (16). This ‘competition score’ is then used to reduce mosaicism in repair outcomes. Microhomology–Predictor does not offer any insights into the level of mosaicism in repair outcomes. In addition, users can scan for only Cas9 NGG sites, whereas MENTHU has been validated using TALENs and offers the ability to search for a wide variety of PAMs.

MENTHU provides several conveniences over FORECasT. The web interface for FORECasT does not allow for automatic analysis of multiple DSB sites along a sequence. It also only supports NGG PAMs; if a non-NGG PAM is of interest, it must be manually specified by its numeric location in the sequence. In contrast, MENTHU scans an input sequence for any targets matching one or more user-specified PAMs or TALENs automatically. In addition, while the FORECasT web interface outputs the predicted repair outcome probabilities for the single specified target site, the downloadable output of the tool consists of a machine-readable file containing a code specifying the deletion, rather than the actual sequence. Thus, while the ability of FORECasT to predict the sequence outcomes for a given DSB is useful, the current web tool is of limited utility for users who wish to locate those sites.

In contrast, inDelphi's web interface is very feature-rich and accepts any Cas9-like PAM. The ‘single’ mode allows users to manually scan for PAM sites in five different cell lines and then outputs the likely mutation probability profile for each. inDelphi outputs additional information including the predicted frameshift probabilities, the predicted distribution of 1 bp insertions and of deletions up to 60 bp in length, the ‘precision’ (the expected proportion of the most prevalent mutation outcome for a given DSB), a ‘microhomology strength’ score, and the frameshift frequency, in addition to detailed information regarding the predicted outcomes.

inDelphi can also be run in batch mode, allowing users to access all of the features in ‘single’ mode for every potential DSB site along an input sequence. Additionally, users can ask inDelphi to recommend gRNAs likely to produce a specified genotypic outcome, which MENTHU does not currently perform. However, this mode is limited to Cas9-like outcomes and pasted input DNA sequences only. inDelphi's ‘gene’ mode offers the ‘batch’ mode treatment for precomputed human (hg38) and mouse (mm10) genes for SpCas9 only. In contrast, MENTHU has been validated in zebrafish models, and can perform expanded scanning within a gene or genomic region of interest based on accession ID, allowing for greater flexibility in target site scanning.

**Figure 6.**
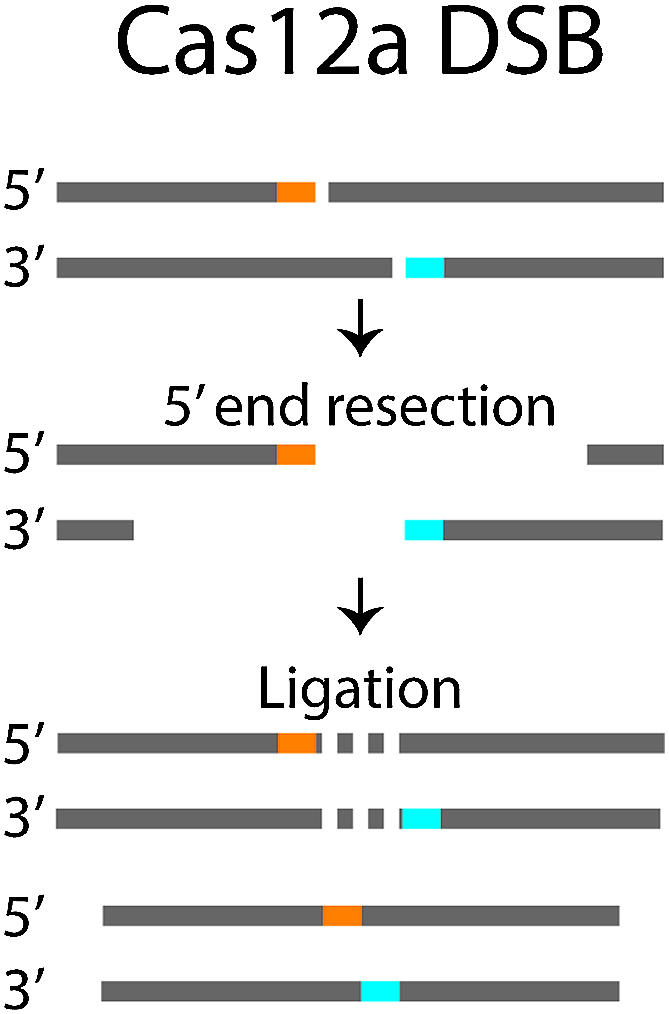
Strategy for handling staggered-cutting nucleases. End-resection operates in a 5′-3′ fashion. 5′ overhangs produced by a staggered-cutting nuclease will be removed during the resection phase. The eliminated sequence in the overhangs is thus unavailable for utilization in MMEJ. We can approximate the microhomologies available for use in MMEJ repair by creating a pseudostring DNA sequence made up of the 5′ strand up until the DSB site (orange) concatenated to the 3′ strand (blue). The 5′ overhangs (dashed lines) are effectively removed. This allows staggered DSBs to be treated identically to blunt DSBs, after the 5′ overhangs are removed from the sequence. The ‘Context’ column within the MENTHU results table (see Figure 5) contains this pseudostring when a staggered-cutting nuclease is chosen.

Unlike FORECasT and inDelphi, MENTHU has been validated for TALEN platforms and supports scanning for PreMA TALEN sites. Additionally, while none of these tools (including MENTHU) have been validated for enzymes that generate staggered-DSBs, such as Cas12a/Cpf1, MENTHU can provide predictions for these sites based on our current understanding of MMEJ repair machinery (Figure 6).

Ultimately, the intended functionality of MENTHU is different from that of inDelphi and FORECasT, which are designed to predict full mutational profiles resulting from specific DSBs. In contrast, MENTHU aims to identify target sites that are likely to result in a particular outcome. Genome engineers will find a more detailed description of editing outcomes in inDelphi and FORECasT, but more accessible targeting recommendations in MENTHU for a wider variety of nucleases and input DNA sequences.

## DISCUSSION

The tools in the GSS are designed to empower researchers to deploy MMEJ-based gene editing, which allows them to focus their efforts on the editing repair outcomes for functional genomics and gene therapy applications. They also enable users to accurately design HMEJ-based targeted gene integration vectors by helping them design oligonucleotides to implement the highly efficient *GeneWeld* strategy for creating knock-in mutations, which has been reported to yield ∼50% germline transmission rates (15).

All tools in the GSS are under active development. Additional GeneWeld plasmid series are nearing completion (J.M. Welker and J.J. Essner, personal communication), and we will add tools for these to GTagHD as they are developed. Work to further improve MENTHU performance in targeting intronic sequences and to validate MENTHU performance for editing with Cas12a systems is underway. We are also using MENTHU to investigate the frequency and occurrence of PreMA alleles (16) in various genomes and producing genome browser tracks to display pre-computed PreMA sites for the entire human genome.

## DATA AVAILABILITY

The GSS is freely available online through www.genesculpt.org.

Each tool is also freely available for download under a GPL v3.0 license at their respective GitHub pages (https://github.com/Dobbs-Lab/GTagHD, https://github.com/Dobbs-Lab/MEDJED, and https://github.com/Dobbs-Lab/MENTHU), which have detailed installation instructions. Each tool can also be downloaded as a Docker image from https://hub.docker.com/r/cmmann/. The GSS was built using a number of third-party R packages: shiny (https://shiny.rstudio.com), shinyjs (https://deanattali.com/shinyjs), stringr (https://cran.r-project.org/web/packages/stringr), stringi (https://cran.r-project.org/web/packages/stringi), plyr (https://cran.r-project.org/web/packages/plyr, (22)), rentrez (https://cran.r-project.org/web/packages/rentrez, (23)), rlist (https://cran.r-project.org/web/packages/rlist), curl (https://cran.r-project.org/web/packages/curl), randomForest (https://cran.r-project.org/web/packages/randomForest, (24)), ggplot2 (https://ggplot2.tidyverse.org, (25)), rhandsontable (https://cran.r-project.org/web/packages/rhandsontable), Biostrings (https://bioconductor.org/packages/release/bioc/html/Biostrings.html), DT (https://rstudio.github.io/DT), jsonlite (https://rdrr.io/cran/jsonlite, (26)), httr (https://cran.r-project.org/web/packages/httr) and Bioconductor (https://bioconductor.org, (27)). All of these packages are freely available, and code to quickly install them is included in GSS installation instructions on GitHub.

Plasmid maps for GeneWeld plasmids are available through GTagHD’s web page. GeneWeld plasmids are available at AddGene: https://www.addgene.org/Jeffrey_Essner/.
